# Supplementary material for: Alternative splicing of latrophilin-3 controls synapse formation
Source: Nature. 2024 Jan 17;626(7997):128–35. doi: 10.1038/s41586-023-06913-9 (PMC10830413; doi:10.1038/s41586-023-06913-9)
Supplement: Supplementary file 2 — Reporting Summary [file 41586_2023_6913_MOESM2_ESM.pdf]

Corresponding author(s): Shuai Wang, Thomas C. Südhof

Last updated by author(s): Oct 19, 2023

Reporting Summary

Nature Portfolio wishes to improve the reproducibility of the work that we publish. This form provides structure for consistency and transparency in reporting. For further information on Nature Portfolio policies, see our Editorial Policies and the Editorial Policy Checklist.

Statistics

For all statistical analyses, confirm that the following items are present in the figure legend, table legend, main text, or Methods section.

n/a	Confirmed	
☐	☒	The exact sample size (*n*) for each experimental group/condition, given as a discrete number and unit of measurement
☐	☒	A statement on whether measurements were taken from distinct samples or whether the same sample was measured repeatedly
☐	☒	The statistical test(s) used AND whether they are one- or two-sided<br>*Only common tests should be described solely by name; describe more complex techniques in the Methods section.*
☒	☐	A description of all covariates tested
☐	☒	A description of any assumptions or corrections, such as tests of normality and adjustment for multiple comparisons
☐	☒	A full description of the statistical parameters including central tendency (e.g. means) or other basic estimates (e.g. regression coefficient) AND variation (e.g. standard deviation) or associated estimates of uncertainty (e.g. confidence intervals)
☐	☒	For null hypothesis testing, the test statistic (e.g. F, t, r) with confidence intervals, effect sizes, degrees of freedom and P value noted<br>*Give P values as exact values whenever suitable.*
☒	☐	For Bayesian analysis, information on the choice of priors and Markov chain Monte Carlo settings
☒	☐	For hierarchical and complex designs, identification of the appropriate level for tests and full reporting of outcomes
☒	☐	Estimates of effect sizes (e.g. Cohen's d, Pearson's r), indicating how they were calculated

Our web collection on statistics for biologists contains articles on many of the points above.

Software and code

Policy information about availability of computer code

Data collection	NIS-Elements AR Nikon (5.21.01)<br>Image Studio LI-COR Biosciences (5.2)<br>Leica Application Suite X (3.7.4.23463)<br>QuantStudio (3)<br>ChromLab (3.3)<br>Snapgene (6.0.2)

Data analysis	QuantStudio Design and Analysis Software (1.4.1)<br>Python (3.8.13)<br>R (4.2.2)<br>Rstudio (2022.12.0.353)<br>Fiji (2.9.0)<br>STAR aligner (2.7.9a)<br>GMAP aligner (2023-04-28)<br>HTSeq (2.0.2)<br>DESeq2 (3.17)<br>CaImAn (1.9.15)<br>Scikit-image (0.20.0)<br>FilFinder (1.7.3)<br>SciPy (1.10.1)<br>Interactive Genome Viewer (v2.16.2)

For manuscripts utilizing custom algorithms or software that are central to the research but not yet described in published literature, software must be made available to editors and reviewers. We strongly encourage code deposition in a community repository (e.g. GitHub). See the Nature Portfolio guidelines for submitting code & software for further information.

Data

Policy information about availability of data

All manuscripts must include a data availability statement. This statement should provide the following information, where applicable:

- Accession codes, unique identifiers, or web links for publicly available datasets
- A description of any restrictions on data availability
- For clinical datasets or third party data, please ensure that the statement adheres to our policy

All raw data supporting the findings of this study are deposited in the Stanford Data Repository (https://purl.stanford.edu/nj297xj2116), except for the high-throughput sequencing data generated from this study which was deposited at Gene Expression Omnibus repository (https://www.ncbi.nlm.nih.gov/geo/query/acc.cgi?acc=gse240791) with accession code GSE240791. Other public datasets analyzed in this work include: Pacbio long-read mRNA sequencing data (accession number PRJNA547800), Cell type-specific sequencing data (accession code: GSE133291, GSE115746), Neuronal activity regulated transcriptome datasets (accession number: GSE175965, GSE104802, GSE152632).

Research involving human participants, their data, or biological material

Policy information about studies with human participants or human data. See also policy information about sex, gender (identity/presentation), and sexual orientation and race, ethnicity and racism.

Reporting on sex and gender	This study did not use human participant/data/material
Reporting on race, ethnicity, or other socially relevant groupings	This study did not use human participant/data/material
Population characteristics	This study did not use human participant/data/material
Recruitment	This study did not use human participant/data/material
Ethics oversight	This study did not use human participant/data/material

Note that full information on the approval of the study protocol must also be provided in the manuscript.

Field-specific reporting

Please select the one below that is the best fit for your research. If you are not sure, read the appropriate sections before making your selection.

☒ Life sciences ☐ Behavioural & social sciences ☐ Ecological, evolutionary & environmental sciences

For a reference copy of the document with all sections, see nature.com/documents/nr-reporting-summary-flat.pdf

Life sciences study design

All studies must disclose on these points even when the disclosure is negative.

Sample size	No statistical methods were used to predetermine sample sizes, but sample sizes in this study are similar to those generally used in the field (ref 4, 8, 9, 18, 24, 25, 28, 36, 48).
Data exclusions	No data were excluded from the analyses.

Replication	All results were independently replicated at least 3 times, as described in the figure legends.
Randomization	Postnatal day 0 mouse pups were randomly chosen for making primary hippocampal cultures. Different litters of animals receive the same injection of lentivirus (control, Lphn3 KO, or Exon31 KO). In culture experiments, cover slips were assgined randomly.
Blinding	Data collection and analysis for TRUPATH/cAMP assay, calcium imaging, synapse puncta and rabies tracing experiments were blinded. All other experiments and analysis were not blinded, because blinding to sample group allocation is not typically relevant to biochemical and high-throughput sequencing analyses.

Reporting for specific materials, systems and methods

We require information from authors about some types of materials, experimental systems and methods used in many studies. Here, indicate whether each material, system or method listed is relevant to your study. If you are not sure if a list item applies to your research, read the appropriate section before selecting a response.

Materials & experimental systems

n/a	Involved in the study	
☐	☒	Antibodies
☐	☒	Eukaryotic cell lines
☒	☐	Palaeontology and archaeology
☐	☒	Animals and other organisms
☒	☐	Clinical data
☒	☐	Dual use research of concern
☒	☐	Plants

Methods

n/a	Involved in the study	
☒	☐	ChIP-seq
☒	☐	Flow cytometry
☒	☐	MRI-based neuroimaging

Antibodies

Antibodies used	Primary antibodies: anti-MAP2 chicken (1:1000, Encor #CPCA-MAP2), anti-vGluT1 guinea pig (1:1000, Millipore #AB5905), anti-Homer1 rabbit (1:1000, Millipore #ABN37), anti-actin mouse(1:1000, Sigma #A1978), anti-Lphn3 mouse (1:1000, SCBT #sc-393576). Secondary antibodies: anti-chicken (1:1000 Alexa 405 ThermoFisher #A48260), anti-rabbit (1:1000 Alexa 488 ThermoFisher #A-11034), anti-guinea pig (1:1000 Alexa 647 ThermoFisher #A-21450), anti-mouse (1:20000, IRDye 800CW, Licor)
Validation	anti-MAP2 is widely used and sold through many vendors, see for example the results of Google Scholar search for CPCA-MAP2 (https://scholar.google.com/scholar?as_sdt=0%2C10&hl=en&inst=5746887945952177237&q=cpca-map2). anti-vGluT1 guinea pig, anti-Homer 1 rabbit and anti-actin were validated in previous paper (doi: 10.1523/JNEUROSCI.0454-20.2020). anti-Lphn3 was validated by the provider (https://www.scbt.com/p/latrophilin-3-antibody-b-6).

Eukaryotic cell lines

Policy information about cell lines and Sex and Gender in Research

Cell line source(s)	HEK 293T (ATCC #CRL-11268)
Authentication	HEK 293T cell was not authenticated since it was directly purchased from ATCC (#CRL-11268)
Mycoplasma contamination	Cell lines were tested negative for mycoplasma contamination using PCR.
Commonly misidentified lines (See ICLAC register)	None of the cell lines used is listed as commonly misidentified

Animals and other research organisms

Policy information about studies involving animals; ARRIVE guidelines recommended for reporting animal research, and Sex and Gender in Research

Laboratory animals	CRISPR/CAS9 knockin mice (Jax stock no: 02485) and C57BL/6 mice (Jax stock no: 000664) were obtained from Jackson Laboratory. P0 pups were used for hippocampal culture experiments; P0, P21 and P35 mice were injected with viruses for the rabies tracing experiments.
Wild animals	This study did not use wild animal.
Reporting on sex	Sex was not considered in this study design.
Field-collected samples	This study did not use field-collected samples

Ethics oversight | All mice described in this study were maintained using established procedures according to protocols approved by the Stanford University Administrative Panel on Laboratory Animal Care.

Note that full information on the approval of the study protocol must also be provided in the manuscript.
