# Supplementary material for: Alternative splicing of latrophilin-3 controls synapse formation
Source: Nature. 2024 Jan 17;626(7997):128–35. doi: 10.1038/s41586-023-06913-9 (PMC10830413; doi:10.1038/s41586-023-06913-9)
Supplement: Supplementary file 1 — Supplementary Figs. 1–3 and Supplementary Tables 1 and 2. [file 41586_2023_6913_MOESM1_ESM.docx]

**Table of Contents:**

**Supplementary Figure 1 | Uncropped immunoblots and RT-PCR gels. a-d**, Immunoblots for Fig.2b (n=6) and ED Fig.9d (n=3). Hippocampal cultured neurons from Cas9 mice were infected with lentivirus expressing indicated gRNA at DIV3 and analyzed at DIV14. Note ΔPBM2 was another gRNA tested for targeting PBM but was not used for this study due to inefficiency in editing PBM. **e-g**, RT-PCR gels for ED Fig. 9e (n=3) by quantifying the abundance of Exon31- and Exon32-containing mRNA, after genomic editing. Hippocampal cultured neurons from Cas9 mice were infected with lentivirus expressing indicated gRNA at DIV3 and analyzed at DIV14 using total RNA converted cDNA. Exon junction regions for Lphn3 Exon31, Lphn3 Exon32, and beta actin (actb) were amplified by PCR. **h-m,** RT-PCR gels for ED Fig. 3 (1^st^ replicate of n=3) by PCR amplifying exon junctions using total RNA converted cDNA, from indicated tissue and developmental age. OB, olfactory bulb; CB, cerebellum; HP, hippocampus; PFC, prefrontal cortex; ST, striatum; RE, retina. Actin was loaded on the same gel (c, d, e, f, g) as loading control, or on a different gel as sample processing control (a, b, h, i, j, k, l, m).

**Supplementary Figure 2 | Uncropped RT-PCR gels for Extended Data Figure 3. a-f,** RT-PCR gels for ED Fig. 3 (2^nd^ and 3^rd^ replicate of n=3) by PCR amplifying exon junctions using total RNA converted cDNA, from indicated tissue and developmental age. OB, olfactory bulb; CB, cerebellum; HP, hippocampus; PFC, prefrontal cortex; ST, striatum; RE, retina. Actin was loaded a different gel as sample processing control.

**Supplementary Figure 3 | Uncropped Coomassie-stained protein gels. a,** Sedimentation assay for Fig. 4b, 4c (n=4). Lphn3 with Exon31, Exon32 or PBM deleted Exon31 was incubated with post-synaptic scaffold proteins GHPS composed of 10µM GKAP, 10µM Homer3, 10µM PSD95, and 10µM Shank3. Sample were sedimented at 5krpm for 5min, after which pellet and supernatant were loaded on the gel. **b,** Sedimentation assay for ED Data Fig. 7a, 7b (n=3). Lphn3 with Exon31 or Exon32 was incubated with GHPS at 120µM total scaffold protein concentration. Sample were sedimented at 5krpm for 5min, after which pellet and supernatant were loaded on the gel. Note the condition using 1krpm was a trial experiment and was not used for this study. **c,** Purified proteins for ED Data Fig.6. Protein fractions from the size exclusion chromatography were shown. Purified proteins were individually centrifuged at 5krpm for 5min to validate the solubility. P, pellet; S, supernatant.

**Supplementary Table 1 | Pairwise multiple comparisons using Tukey’s test for Main Figures.** The test result is labeled by its corresponding figure name. Note gene expression level (Figs. 5a, 5c, 5d) do not follow normal distribution thus were excluded from this analysis.

**Supplementary Table 2 | Pairwise multiple comparisons using Tukey’s test for Extended Data Figures.** The test result is labeled by its corresponding figure name. Note gene expression level (ED Figs. 5c, 10e) do not follow normal distribution thus were excluded from this analysis.
